# Supplementary material for: Splice-Junction-Based Mapping of Alternative Isoforms in the Human Proteome
Source: Cell Rep. Author manuscript; Available in PMC 2020 Jan 15. (PMC6961840; doi:10.1016/j.celrep.2019.11.026)
Supplement: 2 [file NIHMS1546469-supplement-2.zip › DF1/PXD010154/Ovary/Ovary_18_NADSYN1_WDLLAGQPEGLCALNQWALDFEGN.pdf]

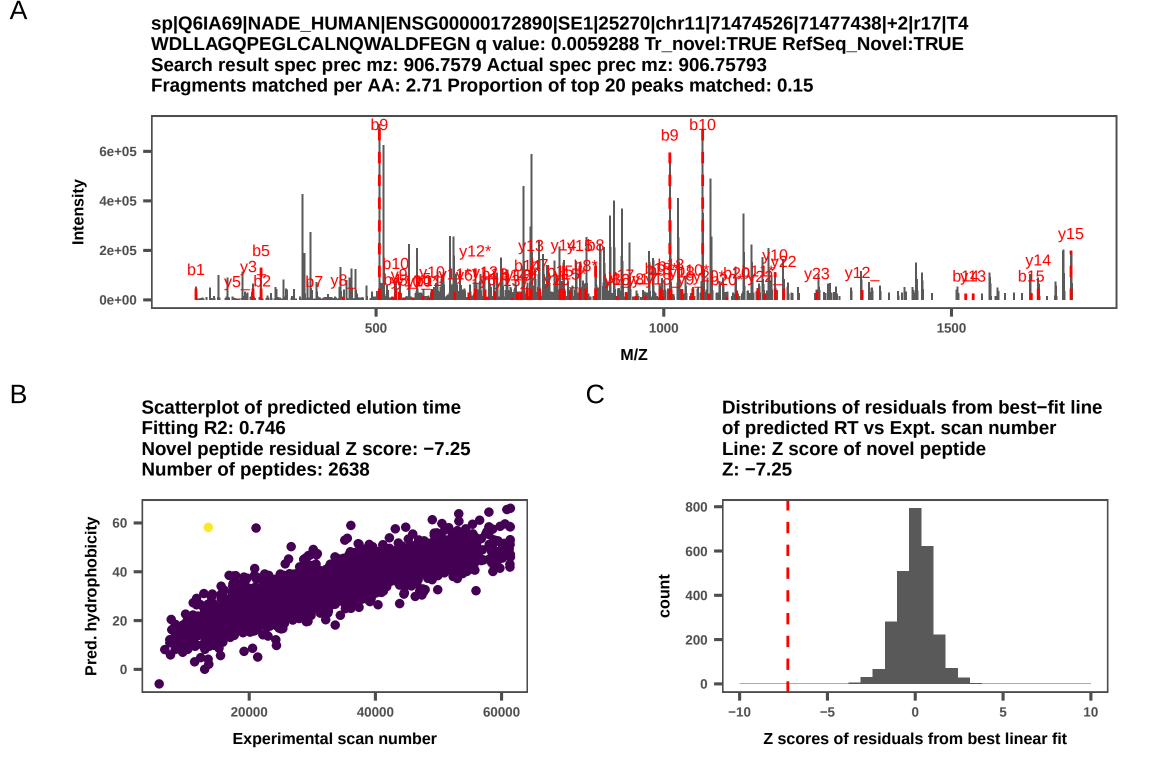
A
sp|Q6IA69|NADE_HUMAN|ENSG00000172890|SE1|25270|chr11|71474526|71477438|+2|r17|T4
WDLLAGQPEGLCALNQWALDFEGN q value: 0.0059288 Tr_novel:TRUE RefSeq_Novel:TRUE
Search result spec prec mz: 906.7579 Actual spec prec mz: 906.75793
Fragments matched per AA: 2.71 Proportion of top 20 peaks matched: 0.15
Intensity
6e+05
4e+05
2e+05
0e+00
500
1000
1500
M/Z
B
Scatterplot of predicted elution time
Fitting R2: 0.746
Novel peptide residual Z score: −7.25
Number of peptides: 2638
Pred. hydrophobicity
60
40
20
0
20000
40000
60000
Experimental scan number
C
Distributions of residuals from best−fit line
of predicted RT vs Expt. scan number
Line: Z score of novel peptide
Z: −7.25
count
800
600
400
200
0
−10
−5
0
5
10
Z scores of residuals from best linear fit
